# Fidgety Parents

**Published:** 1891-07

**Authors:** 


### FIDGETTY PARENTS.

Of all the troublesome people in the world, there are few whose presence is so aggravating to the nerves as the fidgetty. They have such a trick of worrying about the past and the future, and are so uneasy about the present, that a casual meeting and acquaintance with them is unpalatable to persons who are endeavoring to take life, with its ups and downs, with as much calmness as possible. A neighbor's wife is a living example of the influence which the habit of fidgetting may exercise when yielded to. A good mother and a faithful wife is she, yet the cares and duties of the home so completely exhaust her stock of patience and composure that she is unable to meet an emergency in a capable manner. A short time ago, when lifting her baby from its cradle she bumped its head against the wooden hood under which it had beem sleeping, and so upset was she at the mishap that she straightway got a hammer, and smashed the offending article. We may never reach such an uncontrollable condition, yet the lesson to be drawn is good for all to whom the responsibilities of a household are committed. There are so many matters demanding close attention, and the moods of home life are so variable, that unless a cool and even deportment is maintained the little things will rob us of self-control; and, if the heads of the home are fussy, nervous, and easily disturbed, what must be the condition of the rest of the family? In the absence of any reserve power, the slightest derangement of the ordinary routine involves a state of things which amounts almost to a panic. The children generally inherit the characteristics of the parents, and this fact, together with the constant example set them, develops an irritable, uncertain temper that is very uncomfortable to live with. There is friction and unnecessary heat in the household machinery. Family life is uneasy and unsafe, for not only is there an absence of steady, quiet contentment, but there is also the constant danger of some one throwing the house into a ripple of excitement by his fidgetting and restlessness.—*Farm and Home.*

